# Supplementary material for: Mitigating Head Position Bias in Perivascular Fluid Imaging: LD-ALPS, a Novel Method for DTI-ALPS Calculation
Source: NeuroSci. 2025 Oct 7;6(4):101. doi: 10.3390/neurosci6040101 (PMC12550934; doi:10.3390/neurosci6040101)
Supplement: Supplementary file 1 [file neurosci-06-00101-s001.zip › File S1.pdf]

Supplementary Analyses

We compared the different ALPS metrics across the five diagnostic groups (41 Cognitively Normal, 55 at an Early stage of Mild Cognitive Impairment, 32 at a Late stage of Mild Cognitive Impairment, 33 individuals with Alzheimer's Disease, and 10 with Significant Memory Concerns) in the ADNI data we utilized, after exclusion of an outlier subject noted in section 3.3. ANOVA analyses revealed that Unrotated ALPS ($F_{4,166}$ = 2.831, p = .026), vecreg ALPS ($F_{4,166}$ = 2.597, p = .038), and LD-ALPS ($F_{4,166}$ = 3.220, p = .014) displayed significant differences between the diagnostic groups.

ALPS Descriptive statistics between diagnostic groups

	UNROTATED ALPS					VECREG-ALPS					LD-ALPS				
	EMCI	CN	AD	LMCI	SMC	EMCI	CN	AD	LMCI	SMC	EMCI	CN	AD	LMCI	SMC
Valid	55	41	34	32	10	55	41	34	32	10	55	41	34	32	10
Missing	0	0	0	0	0	0	0	0	0	0	0	0	0	0	0
Mean	1.252	1.318	1.214	1.250	1.285	1.306	1.345	1.265	1.305	1.300	1.424	1.489	1.356	1.427	1.471
Std. Deviation	0.177	0.170	0.193	0.151	0.132	0.142	0.133	0.162	0.128	0.102	0.158	0.159	0.166	0.159	0.121
Minimum	0.892	0.949	0.922	1.024	1.104	1.026	1.136	1.008	1.094	1.121	1.009	1.232	1.000	1.172	1.245
Maximum	1.679	1.650	1.932	1.666	1.461	1.723	1.603	1.874	1.584	1.432	1.789	1.805	1.733	1.817	1.629

EMCI = Early stage of Mild Congitive Impairment, CN = Cognitively Normal, AD = Alzheimer's Disease, LMCI = Late stage of Mild Cognitive Impairment, SMC = Significant Memory Concerns
